# Genome-wide identification and expression profiling reveal the regulatory role of U-box E3 ubiquitin ligase genes in strawberry fruit ripening and abiotic stresses resistance

**DOI:** 10.3389/fpls.2023.1171056

**Published:** 2023-03-23

**Authors:** Leiyu Jiang, Yuanxiu Lin, Liangxin Wang, Yuting Peng, Min Yang, Yuyan Jiang, Guoyan Hou, Xiaoyang Liu, Mengyao Li, Yunting Zhang, Yong Zhang, Qing Chen, Yan Wang, Wen He, Xiaorong Wang, Haoru Tang, Ya Luo

**Affiliations:** ^1^ College of Horticulture, Sichuan Agricultural University, Chengdu, China; ^2^ Institute of Pomology & Olericulture, Sichuan Agricultural University, Chengdu, China

**Keywords:** strawberry, U-box ubiquitin ligases, fruit ripening, stresses resistance, gene function

## Abstract

The plant U-box (PUB) proteins are a type of E3 ubiquitin ligases well known for their functions in response to various stresses. They are also related to fruit development and ripening. However, PUB members possess such roles that remain unclear in strawberry. In this study, 155 *PUB* genes were identified in octoploid strawberry and classified into four groups. Their promoters possessed a variety of *cis*-acting elements, most of which are associated with abiotic stresses, followed by phytohormones response and development. Protein-protein interaction analysis suggested that FaU-box members could interact with each other as well as other proteins involved in hormone signaling and stress resistance. Transcriptome-based and RT-qPCR expression analysis revealed the potential involvement of *FaU-box* genes in resistance to stresses and fruit ripening. Of these, *FaU-box98* and *FaU-box136* were positively while *FaU-box52* was negatively related to strawberry ripening. *FaU-box98* comprehensively participated in resistance of ABA, cold, and salt, while *FaU-box83* and *FaU-box136* were broadly associated with drought and salt stresses. *FaU-box18* and *FaU-box52* were ABA-specific; *FaU-box3* was specific to salt stress. In addition, the functional analysis of a randomly selected *FaU-box* (*FaU-box127*) showed that the transient overexpression of *FaU-box127* promoted the ripening of strawberry fruit, along with significant changes in the expression levels of some ripening-related genes and the content of organic acid and soluble sugar. Overall, these findings provided comprehensive information about the *FaU-box* gene family and identified the potential FaU-box members participating in stress resistance and strawberry fruit ripening regulation.

## 1 Introduction

Being one of the important post-translation regulation mechanisms in both eukaryotes and plants, the ubiquitin/26S proteasome system has been well-investigated in recent years (Wang et al., 2018). It plays crucial regulatory roles in various biological processes such as signal transduction (Santner and Estelle, 2010), metabolic regulation, and stress response (Vandereyken et al., 2018), and finally leads to the degradation of specific ubiquitinated substrate proteins (Mukhopadhyay and Riezman, 2007). The ubiquitination of a target protein requires the involvement of a three-step conjugation cascade, which needs the participation of the E1 ubiquitin-activating enzyme, E2 ubiquitin-conjugating enzyme, and E3 ubiquitin ligase (Sharma et al., 2016). Among these three enzymes catalyzing the ubiquitination cascade, E3 ubiquitin ligases are the largest, most diverse group, and have been regarded as the determining factor for the specificity of ubiquitination, due to their ability to recognize the target proteins for modification (Sadanandom et al., 2012). E3 ubiquitin ligases can be classified into nine subfamilies including RING, HECT, U-box, F-box, cullin, BTBT, DDB, RBX, and SKP. The RING, HECT, and U-box E3 types function as single-polypeptide proteins, while the other six E3 types function in multi-subunits complexes. Although U-box E3 ligase is the smallest family among all the E3 ligase subfamilies, it plays several essential roles in plant growth, development, and stress responses (Mao et al., 2022).

The U-box E3 ligases have a signature 70 amino acids-contained U-box domain, which is structurally related to RING-type but lacks the conserved characteristic zinc-integrating cysteine and histidine residues (Yee and Goring, 2009). U-box proteins have been identified in various plants and the gene numbers vary with species. The genome of *Arabidopsis thaliana*, tomato, peach, grape, banana, rice, and soybean have 60, 62, 54, 56, 91, 76, and 120 *U-box* genes respectively (Du et al., 2009; Wang et al., 2016; Hu et al., 2018; Tan et al., 2019; Sharma and Taganna, 2020; Kim et al., 2021). So far, numerous studies have been carried out to investigate the distinct functions of *U-box* genes, and it has been suggested that the U-box proteins extensively participate in organs growth regulation (Li et al., 2021), cell death signaling (Zeng et al., 2008), flowering (Trujillo, 2018), and resistance of stresses (Trujillo, 2018; Chen et al., 2021; Trenner et al., 2022). The apple *U-box* gene *MdPUB23* has been suggested to reduce cold-stresses tolerance (Wang et al., 2022), and *MdPUB24* is proven to be ethylene-activated and promote fruit chlorophyll degradation (Wei et al., 2021). More recently, Tan et al., have demonstrated the *U-box* genes are relevant to ethylene, auxin, and ABA in peach (Tan et al., 2019). It has been also found the *U-box 13*/*43*/*50*/*51* is highly expressed and predicted to have a role in the development and ripening of tomato fruit (Sharma and Taganna, 2020). Moreover, the grapevine U-box E3 ubiquitin ligase *VlPUB38* negatively regulates fruit ripening by facilitating abscisic aldehyde oxidase degradation (Yu et al., 2020). All these results indicate the diverse pivotal roles of *U-box* genes in distinct biological processes including fruit development and ripening. However, the evolutionary aspects, genetic organizations, and functional fate of the *U-box* genes in strawberry are not well understood and needs to be further explored.

Strawberry (*Fragaria* × *ananassa* Duch.) is not only an economical and nutritionally important fruit crop cultivated worldwide, but also an ideally typical model plant for studying the non-climacteric fruit development and ripening. Regulation of strawberry growth, stress resistance, and fruit ripening is important to obtain a high economic value for cultivators and meet the supply-demand of the consumers as well. Previous studies have shown that lots of genes were involved in strawberry stress resistance, such as *WRKY* (Wei et al., 2016), *NAC* (Zhang et al., 2018), and *G6PDH* (Zhang et al., 2020). Studies on strawberry fruit development and ripening have focused on the regulatory roles of ABA (Li et al., 2011), auxin (Mezzetti et al., 2004), and sucrose (Luo et al., 2020). Furthermore, we have previously found the E2 ubiquitin-conjugating enzyme genes play a vital role in strawberry ripening (Li et al., 2022), whereas, whether and which U-box E3 ubiquitin ligase gene members in strawberry are related to stress responses and fruit ripening is still unknown. In this study, we carried out genome-wide characterization analysis of *U-box* genes in cultivated strawberry by concentrating on the gene location, gene structure, evolutionary analysis, functional and protein-protein interaction analysis. The expression profiles of *FaU-box* genes during strawberry fruit development and ripening, and in response to ABA and sucrose treatment were subsequently investigated. Moreover, the responses of *FaU-box* genes under different abiotic stresses in strawberries were explored as well. The results generated here would help understand the putative roles of *U-box* genes in strawberry ripening and stress resistance, and also will enrich the corresponding regulatory network.

## 2 Materials and methods

### 2.1 Identification and comprehensive analysis of *FaU-box* genes

The newest version (v1.0.a2) of the strawberry genome was downloaded from the GDR database (https://www.rosaceae.org) (Sook et al., 2018). The U-box domain seed file (PF04564) was downloaded from the Pfam database (https://pfam.xfam.org) (Finn et al., 2016) and subsequently used to search against the strawberry genome for the candidate *FaU-box* genes by using the HMMsearch program. The completeness of the conserved U-box domain was furtherly confirmed by searching in NCBI conserved domain database (Aron et al., 2011) and SMART dataset. The characteristics of FaU-box proteins including the number of amino acids, putative protein molecular weight (MW), and isoelectric point (pI) were obtained using a perl script. The gene structure was analyzed and visualized by Gene Structure Display Server v.2.0 (http://gsds.cbi.pku.edu.cn/index.php) based on the alignment of their CDSs with corresponding genomic DNA sequences. The MEME online program (http://meme-suite.org/tools/meme) was used to identify the conserved motifs. The upstream 2 Kb sequences of each *FaU-box* gene were retrieved from the corresponding genome as the putative promoter regions. The distribution and types of *cis*-elements in the promoter regions were identified using PlantCARE online software (http://bioinformatics.psb.ugent.be/webtools/plantcare/html/). The subcellular localization was predicted by the WOLF PSORT program (https://wolfpsort.hgc.jp). GO enrichment analysis was carried out by the TopGO R package. Protein-protein interacting network was constructed by the STRING online tool (https://string-db.org).

### 2.2 Phylogenetic and evolutionary analysis of *FaU-box* genes in strawberry

Multiple alignments of FaU-box proteins were performed by Clustal X (v.2.0), and a phylogenetic tree was constructed by MEGA X software (Kumar et al., 2016) with the following parameters: neighbor-joining (NJ) method (Saitou and Nei, 1987), 1,000 bootstrap test replicates. The tree was subsequently beautified by using iTol online tool (https://itol.embl.de/about.cgi) (Letunic and Bork, 2021). Duplication events and the collinear gene pairs were determined using MCScanX software (http://chibba.pgml.uga.edu/mcscan2/). Synteny analysis was carried out by MCScanX and visualized by Circos software. Synonymous (Ks) and non-synonymous (Ka) substitutions per site between gene pairs were subsequently calculated using KaKs Calculator v.1.2 software (Zhang et al., 2006).

### 2.3 Plant materials and treatments

The plants of strawberries were grown in a greenhouse with growth conditions set to 22 ± 2°C, relative humidity 70–90%, and a 14/10 h light/dark regime. Five fruit developmental stages were defined as small green (SG, 7 days after fruit setting [DFS]), large green (LG, 14 DFS), white (W, 20 DFS), partial red (PR, 25 DFS and red surface area more than 1/2), and fully red (FR, 28 DFS and surface area all red) based on days after fruit setting and the color of the receptacle. Fruits were harvested and subsequently ground into powder in liquid nitrogen and stored at –80°C until further use. At least three fruits were mixed as one biological replicate, and three independent biological replicates were collected.

The mixture of ABA and sucrose treatment was the same as previously described (Luo et al., 2020). For ABA treatment, strawberry plants were sprayed with 100 μM ABA, and for cold treatment, the plants were subjected to 4°C and 25°C (used as control) respectively. The leaves were collected at 0, 3, 6, 9, and 12 h for substantial experiments. What’s more, 10% PEG 6000 was used to create hydration conditions for drought treatment, and 200 mM NaCl solution was used to incubate the strawberry plants for salinity treatment. The leaves under salt and drought stresses were collected at 0, 3, 6, 12, and 24 h after treatments. At least 9 plants for each treatment were treated, and leaves from three independent plants were mixed as one biological replicate. All treated samples were immediately frozen in liquid nitrogen for subsequent analysis.

### 2.4 RNA isolation and cDNA synthesis

Total RNA was extracted from samples using the CTAB method. The integrity and purity of RNA were tested by electrophoresed on 1.0% agarose gel and Nanodrop nucleic acid protein instrument. First-strand cDNA was synthesized according to the instruction of the reverse transcription kit purchased from Takara Co. Ltd (Dalian, China).

### 2.5 Expression analysis of *FaU-box* genes

The RNAseq-based expression levels of *FaU-box* genes during strawberry fruit developmental stages and ABA and sucrose treatment were retrieved from our previous study (Luo et al., 2020). The transcript abundance of *FaU-box* genes was reflected by the FPKM value. RT-qPCR-based expression analysis was carried out using SYBR Green Premix Ex TaqTM (Takara, Japan) on a CFX96 RT-qPCR system (Bio-Rad, USA) in triplicate of each sample. Fluorescence was recorded at the end of the annealing step of each cycle. A melting curve was inserted, ramping from 65°C to 95°C (increment 0.5°C/5 s) after the final cycle. The relative expression of *FaU-box* genes was calculated using the 2^-ΔΔCt^ method (Livak and Schmittgen, 2001). *FaActin* (accession: LC017712) and 26-18S interspacer RNA (Jiang et al., 2022) were used as the reference genes to normalize the raw expression data. All data were represented by the mean ± standard deviation (SD) of three independent biological replicates. All primers used for RT-qPCR in the present study were listed in **Supplementary Table S9**.

### 2.6 Statistical analysis

Data were analyzed by IBM SPSS Statistics software (version 26.0). One-way ANOVA analysis was conducted, the significant differences between multiple groups were calculated by the LSD multiple comparison method, and pairwise comparison was tested by using Turkey’s test method. A *P* value ≤ 0.05 was considered as a significant difference.

## 3 Results

### 3.1 Identification and characteristics of the U-box gene family in strawberry

**Figure 1:**
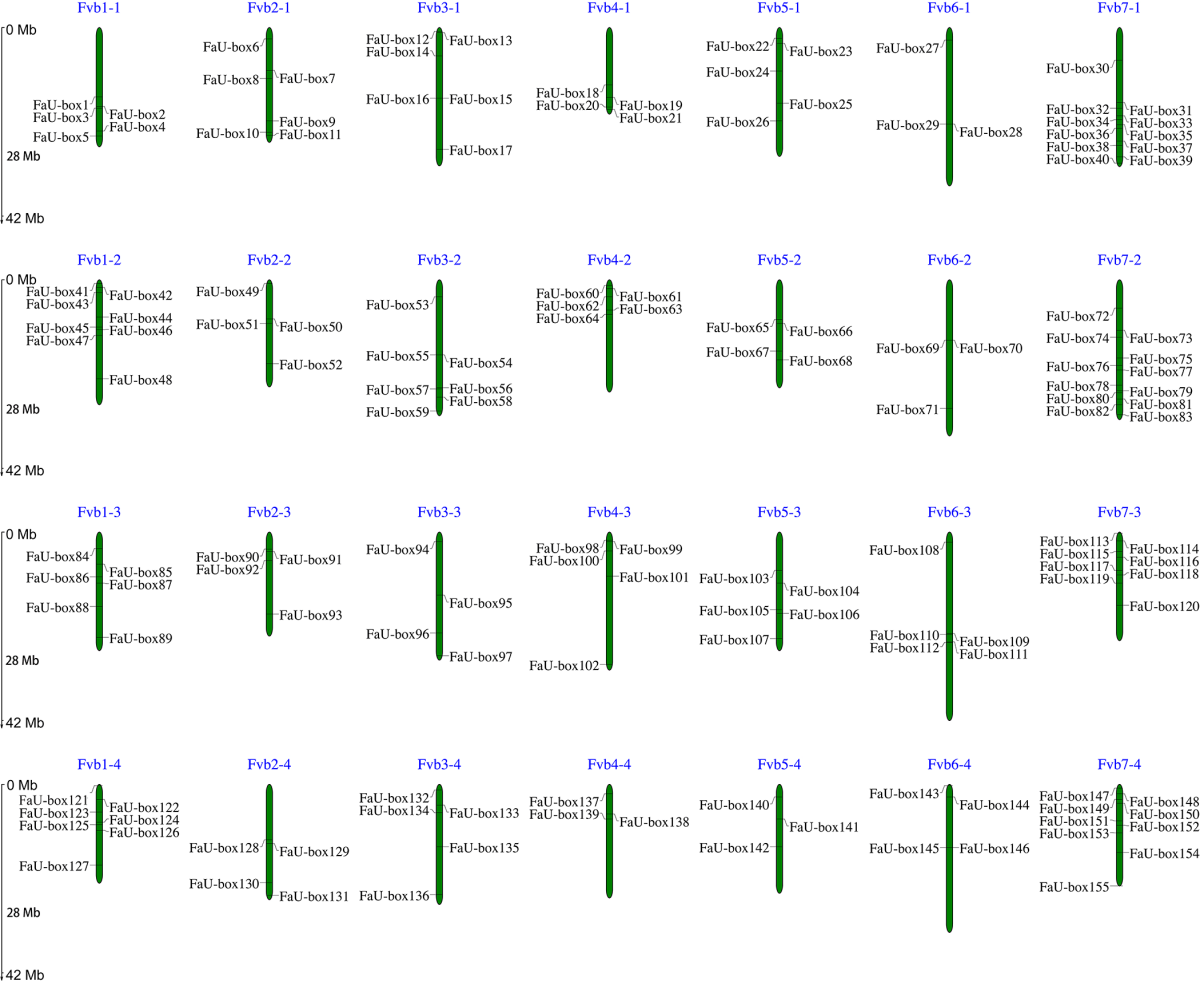
Distribution and chromosomal localization of FaU-box genes. The green columns represented the octoploid strawberry chromosomes. The blue letters at the top of each chromosome indicated the chromosome number.

A total of 155 *FaU-box* genes were identified by using HMMER and SMART tools (**Supplementary Table S1**). According to their distribution orders on the strawberry chromosomes, all the identified *FaU-box* genes were renamed from *FaU-box1* to *FaU-box155* (**Figure 1**). As the result showed, although they were unevenly located on the 28 chromosomes, it seemed like there was a location preference on chromosome 7 in the 4 subgenomes of cultivated strawberries. The gene numbers per chromosome ranged from 3 to 12, with a maximum of 12 on chromosome 7 from the second subgenome (Fvb7-2), and a minimum of 3 on chromosomes 4 and 5 from the fourth subgenome (Fvb4-4 and Fvb5-4) as well as chromosome 6 from the first and second subgenomes (Fvb6-1 and Fvb6-2). The result of the physicochemical properties analysis (**Supplementary Table S1**) suggested that 155 FaU-box proteins ranged from 116 to 1476 in length, having a calculated molecular weight of 13 to 164 kDa. The isoelectric points varied from 4.76 to 9.31, and about 71 FaU-box proteins had an isoelectric point above 7. From the subcellular localization result, it was evident that most FaU-box proteins might be located in the nucleus, while the remaining were predicted to be found in the cytosol, plasma, chloroplast, and mitochondrion.

### 3.2 Gene structure, motif analysis, and gene ontology functional annotations of FaU-box genes

**Figure 2:**
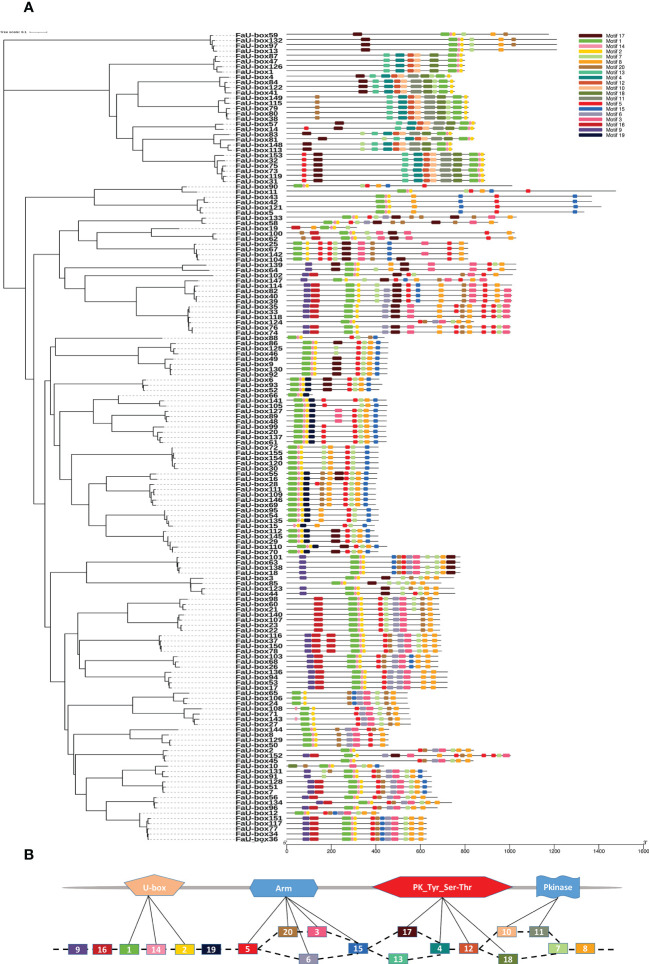
Distribution and location of conserved motifs of FaU-box proteins. **(A)**, Phylogenetic relationship and distribution of 20 conserved motifs analyzed by MEME online tool in the 155 FaU-box proteins. **(B)**, Annotation of the 20 conserved motifs based on the Pfam database.

The exon numbers in the *FaU-box* genes varied from 1 to 15 (**Supplementary Figure S1**). Among them, the *FaU-box11*, *FaU-box42*, and *FaU-box43* contained the most exons (15), followed by *FaU-box5* and *FaU-box121*, possessing 14 exons, while a total of 67 *FaU-box* genes (43.2%) contained only a single exon. In addition, the motif analysis reflected that 20 conserved motifs were found in the amino acids sequence of FaU-box proteins (**Figure 2A**). As shown, all FaU-box proteins contained the core motifs (motifs 1, 2, and 14), which were annotated as U-box domains (**Figure 2B**). Motifs 20, 5, 6, 3, and 15 were ARM conventional motifs, the 4, 12, 17, and 18 were PK_Tyr_Ser-Thr motifs, while the PK (pkinase) motifs were 10 and 11. Motif 8 encoded a GTPase-binding Domain, motif 16 was an activator interacting domain, while the others were unknown.

Gene ontology (GO) enrichment analysis of FaU-box genes was conducted to predict the biological and molecular functions in cellular metabolism. According to the result (**Supplementary Table S2**, **Figure S2**), 10 GO function terms were significantly enriched including 8 molecular function (MF) entries and 2 biological process (BP) entries. The GO terms “catalytic activity”, “ubiquitin-protein”, and “transferase activity” were described as the largest terms with 154 genes. The terms namely “protein kinase activity”, “phosphotransferase activity” as well as “response to stress” and “response to stimulus” were also enriched, suggesting the diverse potential roles of FaU-box genes in these processes. However, no cellular component entries were enriched.

### 3.3 Phylogenetic, evolution, and synteny analysis of the FaU-box members

**Figure 3:**
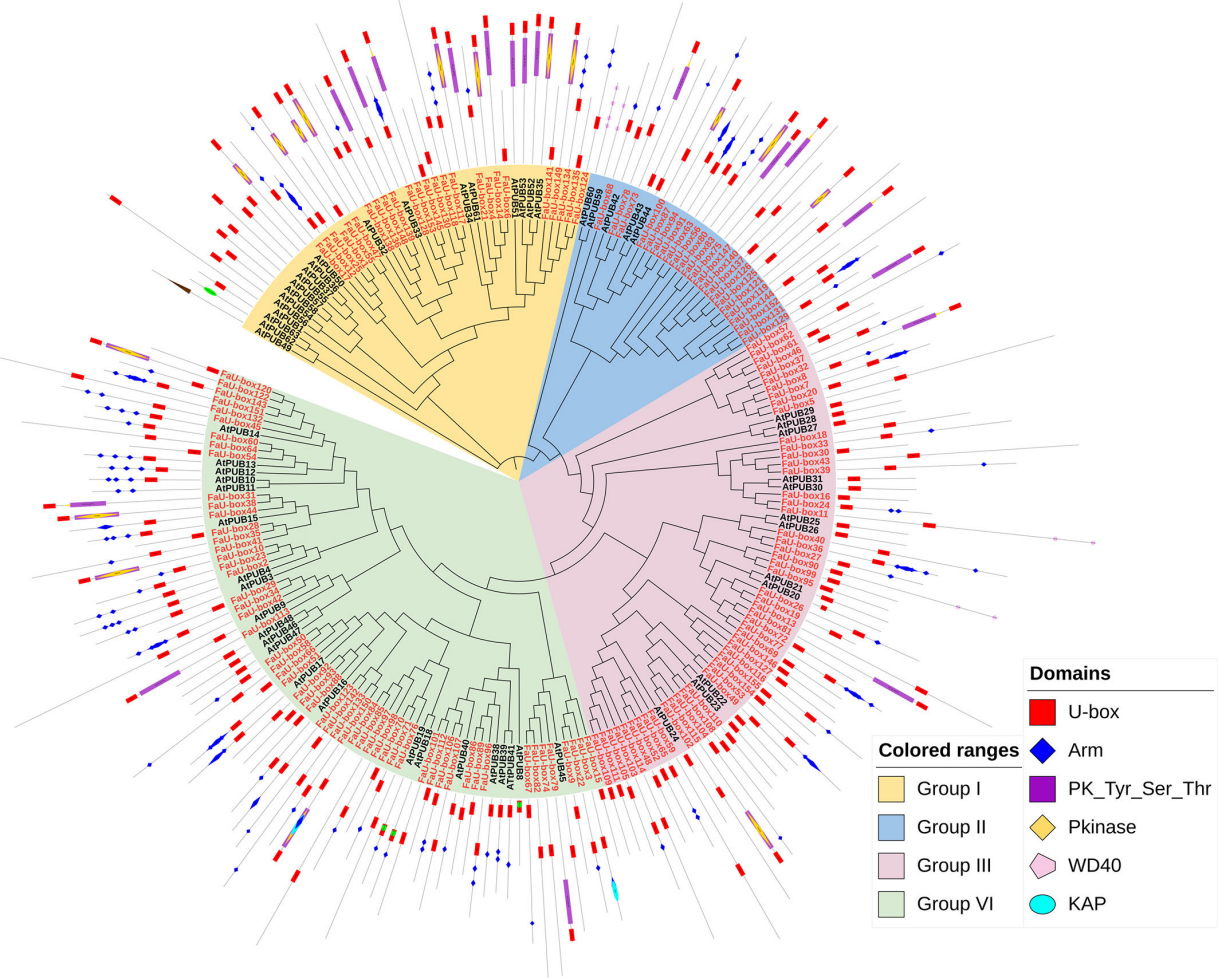
Phylogenetic relationship and conserved domains of U-box members in strawberry and Arabidopsis. The FaU-box genes nodes identified in this study were red-colored, while the AtPUB nodes from Arabidopsis were black.

A phylogenetic tree (**Figure 3**) was constructed based on the U-box proteins identified from strawberry and *Arabidopsis thaliana* (63 members), to investigate the evolutionary relationship of *U-box* genes. It was shown, the 155 *FaU-box* genes were clustered into 4 groups. Group IV contained the most *FaU-box* members (57), followed by groups III (50), I (25), and II (23). Moreover, the conserved protein domains analysis showed that the Arm, PK_Tyr_Ser_Thr, PKinase, KAP, and WD40 domains were also observed in multiple FaU-box proteins (**Figure 3**). Although the domains were not clearly classified with the phylogenetic groups, it could be found that group I mainly contained U-box, Arm, and PK_Tyr_Ser_Thr domains, group II mainly included the U-box and other domains, group III mainly conferred only U-box domain, while group IV mainly had U-box and Arm domains.

**Figure 4:**
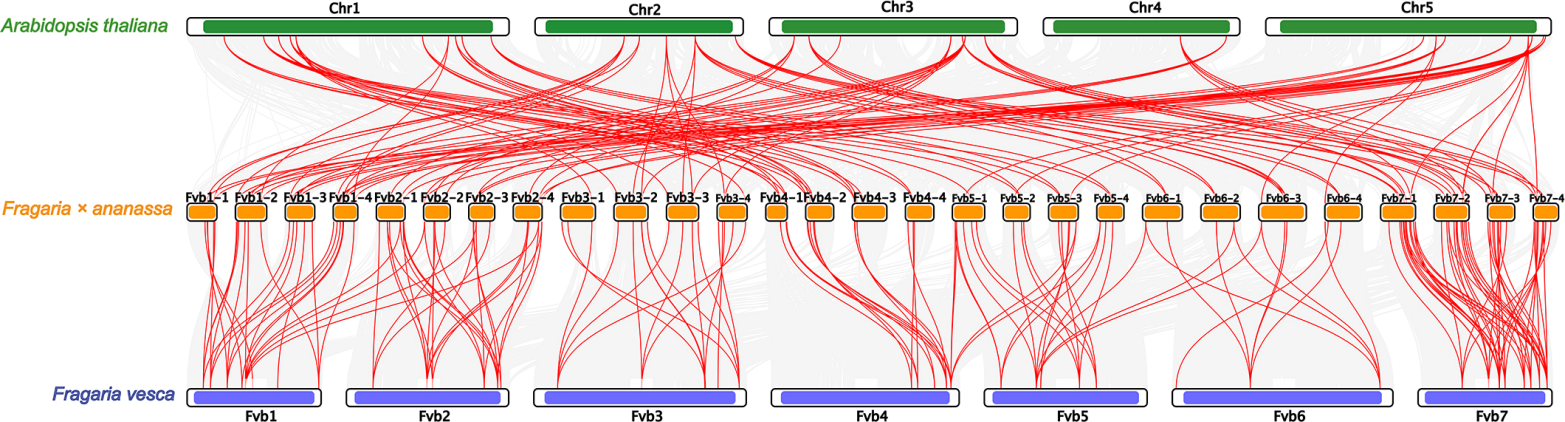
Collinearity analysis of *U-box* genes among *Arabidopsis thaliana*, *Fragaria × ananassa*, and *Fragaria vesca* genomes. Grey lines indicated collinear blocks within the three genomes, while the red lines represented collinear *U-box* gene pairs. The green, yellow, and blue columns indicated the chromosomes from *Arabidopsis thaliana*, *Fragaria × ananassa*, and *Fragaria vesca* genomes respectively. Chromosomes number were displayed at the side of chromosomes.

Collinearity analysis exhibited that 247 *FaU-box* gene pairs in *Fragaria × ananassa* were collinear (**Supplementary Figure S3**, **Table S3**). Most of the *FaU-box* pairs co-located in the same or adjacent chromosome were fragmental duplications. To illustrate the evolutionary relationship of *FaU-box* genes, a collinearity analysis among *Arabidopsis*, woodland strawberry (*Fragaria vesca*), and cultivated strawberry (*Fragaria × ananassa*) was performed (**Figure 4**). The result revealed 138 *FaU-box*, 32 *AtU-box*, and 41 *FvU-box* genes were involved to form a total of 276 collinear pairs (**Supplementary Table S3**). In particular, 113 pairs were identified between *Arabidopsis* and cultivated strawberry, and 163 found between woodland strawberry and cultivated strawberry were highlighted in **Figure 4**. Subsequently, the origins of duplication events of *FaU-box* genes in strawberries were tested using the MCScanX package. As a result, only two types of duplication events were detected, including whole genome duplication or segmental (WGD/segmental) and dispersed (**Supplementary Table S1**). While only *FaU-box101*, *FaU-box12*, *FaU-box155*, *FaU-box19*, and *FaU-box66* were duplicated from a dispersed duplication event, most *FaU-box* genes were duplicated by WGD/segmental event. Additionally, the number of non-synonymous substitutions per non-synonymous sites (Ka), the number of synonymous substitutions per synonymous sites (Ks), and the Ka/Ks ratio for paralogous gene pairs of *FaU-box* genes were calculated. The results suggested that the Ka and Ks ranged from 0 to 0.6 and 0 to 4.01 respectively. The ratio of Ka/Ks ranged from 0 to 1.50, most of the *FaU-box* pairs have a Ka/Ks ratio less than one, while only three pairs have a Ka/Ks ratio greater than one, indicating they are under positive selection during the evolution process (**Supplementary Table S4**).

### 3.4 Cis-acting elements in putative promoter regions of FaU-box genes

The *cis*-elements in the promoter regions of *FaU-box* genes were analyzed. According to the results, in addition to the TATA-box and CAAT-box which are the basic core promoter elements in higher plants, the 155 *FaU-box* promoters included a total of 5,690 *cis*-elements belonging to 44 types (**Supplementary Table S5**). Among them, the most *cis-*elements were associated with abiotic and biotic stress response, followed by plant growth and development, and phytohormone response (**Supplementary Figure S4**). In particular, a total of 130, 111, 114, and 102 *FaU-box* promoters were found to contain G-box, Box 4, GT1-motif, and TCT-motif that were involved in light response during plant growth and development respectively. Besides, many other regulatory cis-elements involved in light responsive (I-box, GATA-motif, and AE-box), endosperm-specific (AACA-motif), root-specific (motif I) as well as seed-specific regulation (RY-element) were found in the *FaU-box* promoters during plant growth and development. Among the phytohormones responsive elements, 405 ABA-responsive elements (ABRE), 628 methyl jasmonate response elements (314 CGTCA-motif and 314 TGACG-motif), 97 salicylic acid-responsive elements (4 SARE and 94 TCA-element), 110 auxin-responsive elements (26 AuxRR-core and 84 TGA-element), and 138 gibberellin-responsive elements (75 P-box, 42 GARE, and 21 TATC-box) were found in multiple *FaU-box* promoters. Moreover, drought response elements (MYB and MYC), stress response elements (STRE), and low-temperature response (LTR) were also detected in the promoter regions. It was worth noting that 9 *FaU-box* promoters contained MYB binding site (MBSI) involved in flavonoid biosynthetic genes regulation (**Supplementary Table S5**). Specifically, most *FaU-box* promoters contained 21-30 *cis*-elements (**Supplementary Figure S4**), among which, *FaU-box102* contained the largest number of elements (64), followed by *FaU-box64* (57), *FaU-box93* (57), and *FaU-box24* (54). Furthermore, the types of elements contained in each *FaU-box* varied (**Supplementary Figure S4**). Most *FaU-box* promoters included 18 types of elements, followed by 17, 19, 16, and 22 types. *FaU-box93* contained the most type’s elements (27), while *FaU-box112* only contained 5 types of elements in promoter regions. The distribution and order of *cis*-elements in the promoter regions were shown in **Supplementary Figure S5**.

### 3.5 Protein-protein interaction network analysis of FaU-box members

**Figure 5:**
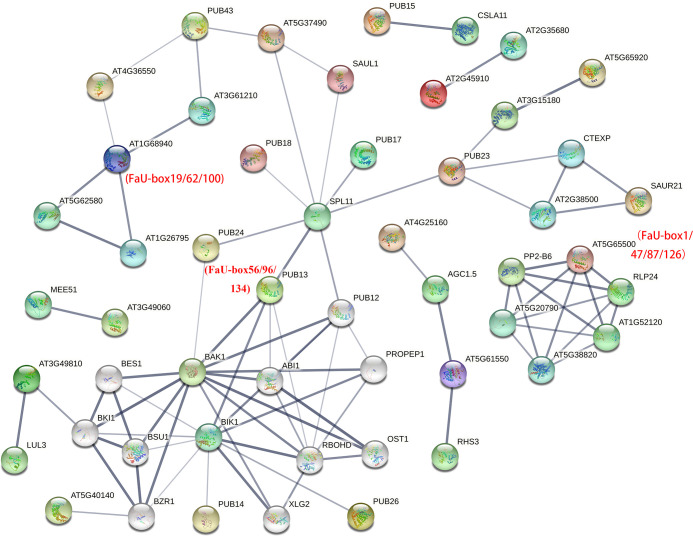
Protein-protein interaction analysis of FaU-box members. The analysis was conducted by the STRING online tool, and the annotation of each protein was shown in **Supplementary Table S7**. Different colored balls indicated interacting proteins, and the protein structures were shown inside each ball. The lines represented the interaction and the line thickness indicated the strength of data support.

To further identify the putative interacting proteins, the interaction network analysis of FaU-box members was performed based on their orthologues in Arabidopsis (AtPUB proteins). By detecting with orthofinder software, 107 out of 155 (69%) FaU-box have orthologues in Arabidopsis (**Supplementary Table S6**). As illustrated in **Supplementary Table S7** and **Figure 5**, various FaU-box members showed interaction with hormone- and stresses-related proteins. For instance, AtPUB13 (orthologues of FaU-box56/96/134) showed interactions with the key component and repressor of ABA (ABI1), brassinosteroid insensitive 1 (BAK1), calcium-dependent NADPH oxidase (ROBHD), serine/threonine kinase BIK1, and squamosal promoter-binding-like protein 11 (SPL11). What’s more, it was also predicted that FaU-box members interacted with each other. As an example, the AtPUB42 (AT1g68940, homologues of FaU-box19/62/100) displayed interaction with AtPUB50 (AT4g36550, homologues of FaU-box3/44/85/123), which also has an interaction with AtPUB43 (AT1g76390, homologues of FaU-box25/67/104/142).

### 3.6 Expression analysis of FaU-box genes during strawberry development and ripening

**Figure 6:**
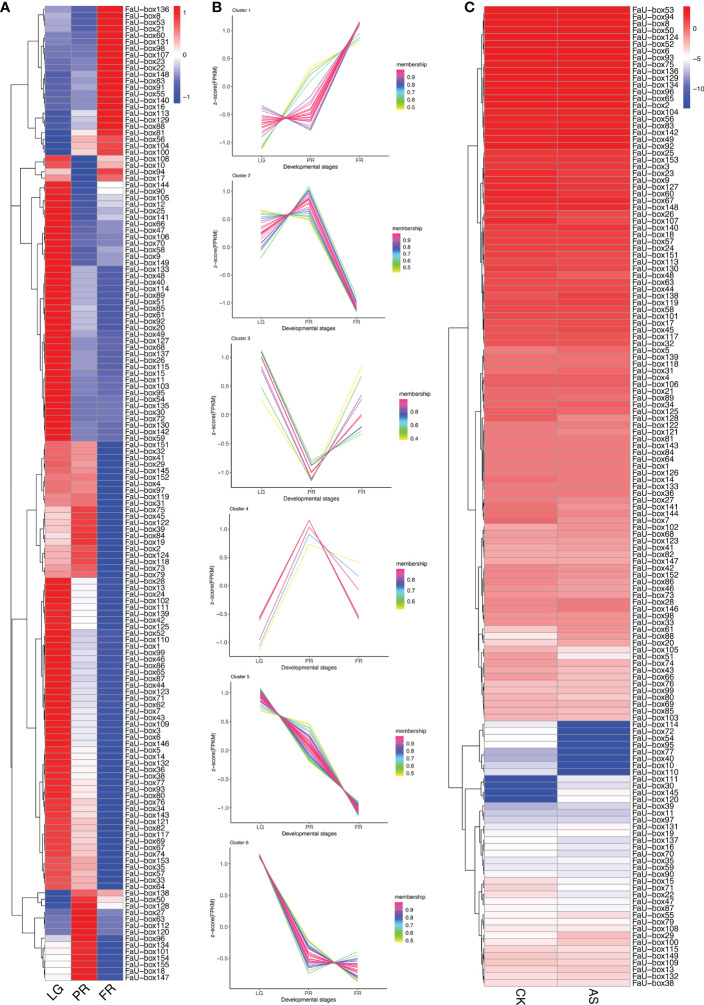
Expression analysis of *FaU-box* genes during fruit development and under ABA and sucrose treatment. **(A)**, RNAseq retrieved expression during fruit development and ripening. **(B)**, Clusters of *FaU-box* genes expression trend during different developmental stages. **(C)**, Transcript abundance of *FaU-box* genes under the exogenous ABA and sucrose treatment. The heatmap represented the log2 value of FPKM. LG, large green; PR, partial red; FR, fully red. AS, ABA, and sucrose treatment.

To elucidate their potential roles in strawberry fruit ripening, the RNAseq-based expression patterns of 155 *FaU-box* genes were assessed. As displayed in **Figure 6A**, 150 out of 155 *FaU-box* genes were expressed with distinct patterns during fruit development. In general, most *FaU-box* genes were highly expressed in the large green (LG) stage, while barely expressed in the partial red (PR) stage. Based on their expression trend, all the 150 expressed *FaU-box* genes were clarified into 6 clusters (**Figure 6B**) in different developmental stages. Overall, clusters 5, 6, and 2 showed similar decline expression from LG to PR stage during the fruit development and ripening. Among them, cluster 5 included the most genes with a number of 47, followed by clusters 6, and 2 with a number of 37 and 26 *FaU-box* genes separately. By contrast, the expression of 23 *FaU-box* genes included in cluster 1 gradually increased during fruit ripening. Cluster 3 and Cluster 4 consisted of 10 and 7 *FaU-box* genes respectively. Genes involved in cluster 3 dramatically decreased from LG to PR stage, while sharply increased thereafter from PR to fully red (FR) stage. However, an opposite change in expression during fruit ripening was observed for the genes included in cluster 4.

To further investigate the potential regulatory mechanism of *FaU-box* genes in strawberry ripening, the transcript abundance in response to ABA and sucrose was also determined. According to the results (**Figure 6C**, **Supplementary Table S8**), most *FaU-box* genes were ABA and sucrose non-responsive, which displayed similar transcript abundance under ABA and sucrose treatment (AS) compared with normal conditions (CK). While some *FaU-box* genes were ABA and sucrose responsive, which were either induced or repressed. For instance, 4 *FaU-box* genes including *FaU-box111*, *FaU-box30*, *FaU-box145*, and *FaU-box120* were barely expressed in CK, while expressed under the ABA and sucrose treatment to a certain extent. On the contrary, several FaU-box genes such as *FaU-box114*, *FaU-box72*, and *FaU-box54* were largely inhibited by ABA and sucrose treatment.

### 3.7 Validation of FaU-box gene expression by real-time quantitative PCR

**Figure 7:**
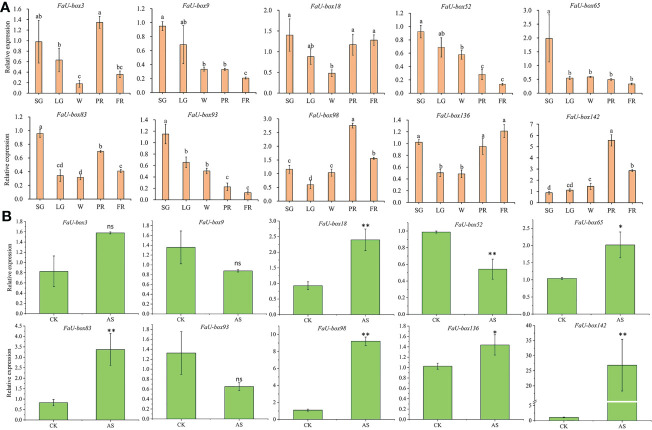
Validation of select *FaU-box* genes by RT-qPCR method. **(A)**, RT-qPCR expression analysis of 10 *FaU-box* genes during fruit development and ripening. SG, small green; LG, large green; W, white stage; PR, partial red; FR, fully red. **(B)**, RT-qPCR expression analysis of *FaU-box* genes in response to ABA and sucrose treatment. AS, ABA, and sucrose treatment. The letters on the top of the bars suggested a significant difference at *p ≤* 0.05 level. One asterisk and two asterisks represented significant differences at *p ≤* 0.05 and *p ≤* 0.01 levels respectively. ns, non-significance.

Real-time quantitative PCR (RT-qPCR) experiments were carried out to validate the accuracy of the transcriptome data. A total of 10 *FaU-box* genes were selected to quantify the expression by using the RT-qPCR method, based on their regular expression patterns and comparative high expression levels during the fruit ripening. The 10 selected *FaU-box* genes were comprised of 3 from cluster 1 (*FaU-box83*, *FaU-box98*, and *FaU-box136*), one from cluster 2 (*FaU-box18*), three from cluster 5 (*FaU-box3*, *FaU-box65*, and *FaU-box93*), and three from cluster 6 (*FaU-box9*, *FaU-box52*, and *FaU-box142*). Consistent with the transcriptome data, the RT-qPCR results showed that the expression of *FaU-box9*, *FaU-box52*, and *FaU-box93* gradually decreased during fruit development and ripening (**Figure 7A**). Likewise, *FaU-box65* expression peaked at the small green (SG) stage, and sharply decreased at LG, a gradual decrease pattern was observed thereafter. Whereas *FaU-box83*, *FaU-box98*, and *FaU-box136* exhibited high expression levels at the SG stage, followed by a general increase trend from LG to PR stages, and a slight decrease at the PR stage. Similarly, the expression level of *FaU-box142* gradually increased from the SG stage and reached the peak at the PR stage, then slightly decreased at the PR stage. The expression of *FaU-box3* and *FaU-box18* decreased from SG to white (W) stage firstly and then increased at the PR stage, eventually, their expressions decreased again or maintained at a relatively stable level respectively. In addition, except for the *FaU-box98* and *FaU-box142*, most of the other selected *FaU-box* genes showed similar results in RT-qPCR assays (**Figure 7B**) and transcriptome-based expression analysis. For example, the expressions of *FaU-box9*, *FaU-box52*, and *FaU-box93* were slightly decreased while *FaU-box18*, *FaU-box 65*, and *FaU-box136* exhibited opposite trends under ABA and sucrose treatment compared to the CK group. Overall, the results of RT-qPCR and transcriptome analysis were consistent, indicating that *FaU-box* genes were involved in fruit development and ripening of strawberries.

### 3.8 Expression analysis of FaU-box genes in response to ABA and abiotic stresses

**Figure 8:**
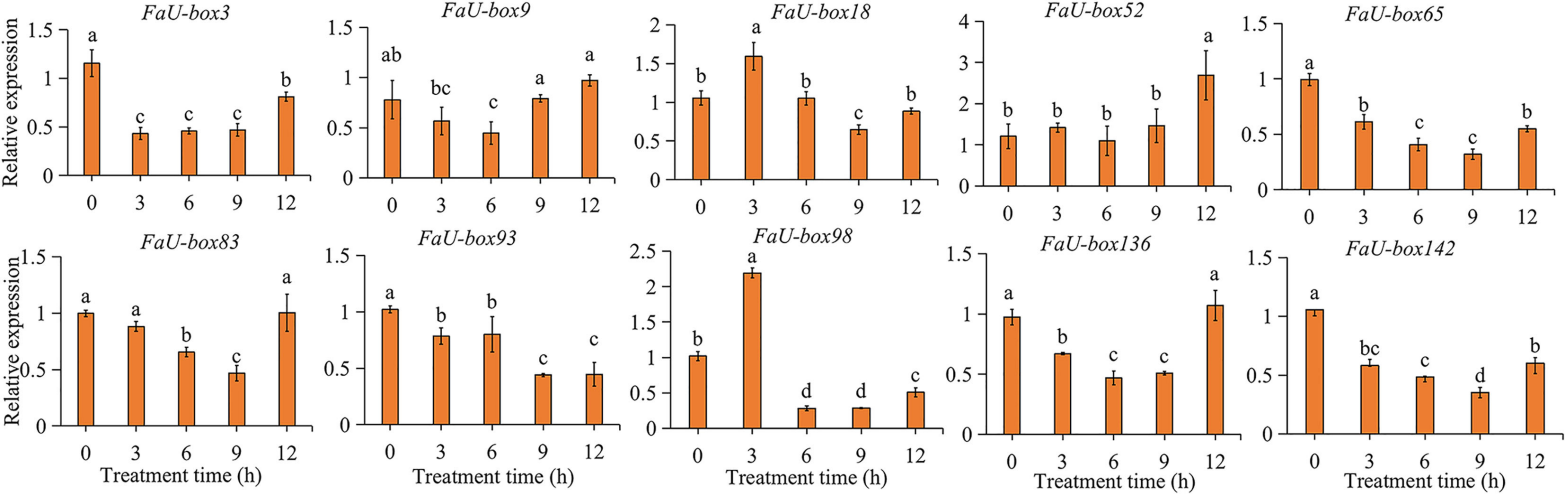
Relative expression levels of selected *FaU-box* genes under the exogenous ABA treatment. Data were represented by mean ± standard deviation. The letters on the top of the bars suggested a significant difference at *P* ≤ 0.05 level.

ABA is not only one of the key regulators of strawberry fruit ripening, but also a stress hormone. The responsiveness of *FaU-box* genes under ABA treatment was estimated in this study. The results (**Figure 8**) showed us, all the selected 10 *FaU-box* genes were differentially responsive to ABA treatment. In detail, the expression of *FaU-box3*, *FaU-box65, FaU-box93*, and *FaU-box142* decreased in response to ABA. On the contrary, the *FaU-box18* and *FaU-box98* were significantly induced by ABA treatment at 3 h. The transcript level of *FaU-box52* showed no obvious change until 12 h after ABA treatment. The expression levels of *FaU-box9*, *FaU-box83*, and *FaU-box136* were firstly reduced by ABA treatment and then increased to a similar level with the CK samples (0 h).

**Figure 9:**
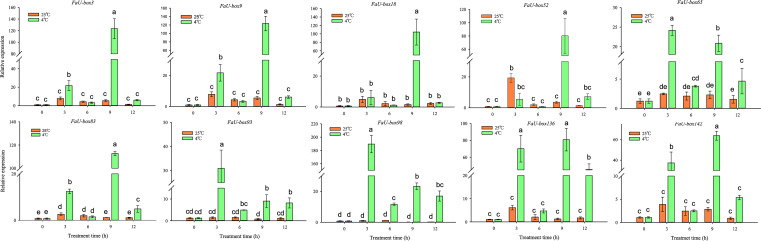
Expression profiles of *FaU-box* genes in response to low-temperature. Data were represented by mean ± standard deviation. The different letters on the bars indicated the significant difference at *P* ≤ 0.05 level.

*U-box* genes have been extensively reported to be involved in various abiotic stresses. To explore the potential functions of *FaU-box* genes in resistance to stress in strawberries, we detected the expression levels of *FaU-box* genes in strawberry seedling samples subjected to three different stress treatments including low-temperature, drought, and salt. The 10 *FaU-box* genes selected for RT-qPCR validation were also used to estimate their expression under different stresses. It was found that 10 *FaU-box* genes differentially respond to low-temperature (4°C) compared to the control (25°C) (**Figure 9**). The expression of *FaU-box93* and *FaU-box98* largely increased at 3 h low-temperature treatment and showed a significantly higher level than the control although it decreased thereafter. The expression of *FaU-box3*, *FaU-box9*, and *FaU-box142* exhibited similar patterns, which showed a significant increase at only 3 h and 9 h under the low-temperature treatment. In addition, the expression of *FaU-box65*, *FaU-box83*, *FaU-box93*, and *FaU-box136* were largely up-regulated at 3 h, 9 h, and 12 h after low-temperature treatment. Whereas no obvious difference was observed at the first 6 h treatment, significant up-regulation was found for the expression of *FaU-box52* after 9 h low-temperature treatment.

**Figure 10:**
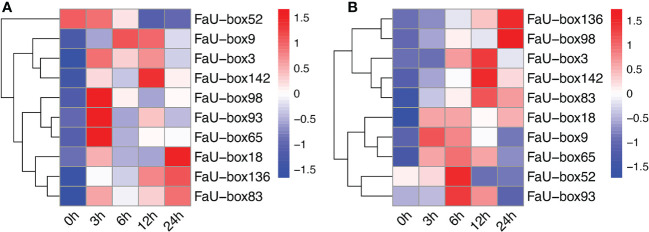
Expression profiles of *FaU-box* genes in response to abiotic stresses. **(A)**, Expression analysis of selected *FaU-box* genes under drought stress. **(B)**, Expression profiles of *FaU-box* genes under salt treatment.

Under drought stress (**Figure 10A**), we found that all of the selected 10 *FaU-box* genes were rapidly up-regulated expressed, except for *FaU-box52*, whose expression exhibited no obvious change at the beginning of treatment (3 h), and gradually decreased to a lower level than untreated thereafter. Furthermore, except for the *FaU-box9*, the expression levels of other *FaU-box* genes were sharply decreased from 3 to 6 h treatment. Then, a continuous decrease for *FaU-box9* and *FaU-box52* expression, while an increase for other *FaU-box* genes at 9 h treatment was observed. Thereafter, substantially continuously increase (*FaU-box18*, *FaU-box83*, and *FaU-box136*), slightly decreasing but still higher than untreated samples (*FaU-box3*, FaU*-box9*, *FaU-box93*, *FaU-box98*, and *FaU-box142*) or no obvious change (*FaU-box65*) were found respectively.

In addition, it was found that all of the detected *FaU-box* genes were responsive to salt treatment (**Figure 10B**), the response time differed with genes however. For example, the expression levels of *FaU-box9*, *FaU-box18*, *FaU-box52*, and *FaU-box65* immediately increased at 3h treatment of salt stress, while the *FaU-box98* and *FaU-box136* moderately increased after 3h and reached their peak at 24h salt treatment. Likewise, the expression of *FaU-box3*, *FaU-box83*, and *FaU-box142* gradually increased and peaked at 12 h, then a slight decrease occurred at 24 h under salt treatment. Furthermore, inhibition of *FaU-box* gene expression was observed for *FaU-box9*, *FaU-box52*, *FaU-box65*, and *FaU-box93* under a long-time (12-24 h) salt treatment.

### 3.9 Transient overexpression of FaU-box127 in strawberry fruit

**Figure 11:**
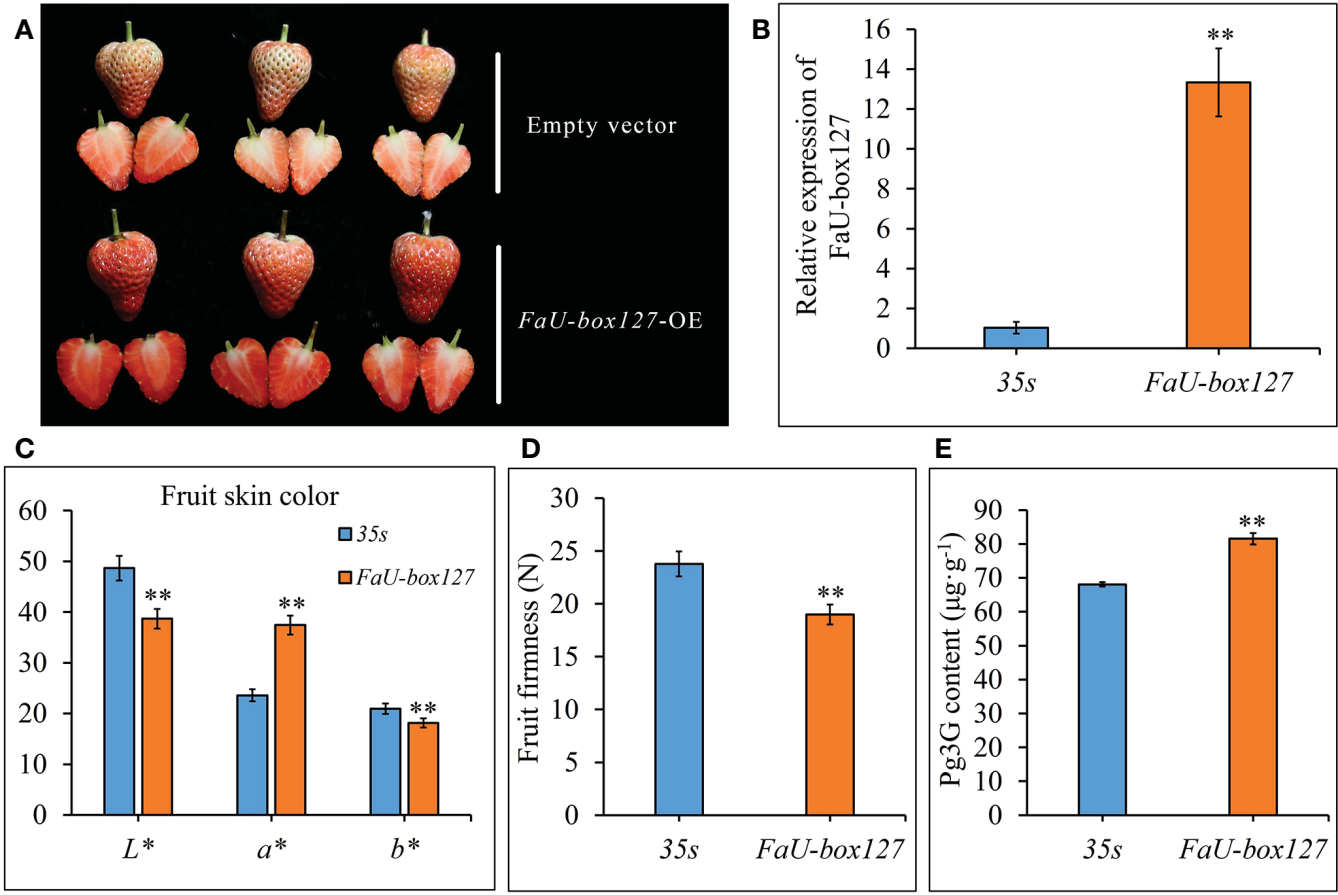
Overexpression of *FaU-box127* in strawberry fruit. **(A)**, the phenotypes of control fruit infiltrated with empty vector and *FaU-box127*-overexpressed fruit infiltrated with agrobacteria harboring *FaU-box127*-overexpressing recombined plasmid. **(B)**, relative expression of *FaU-box127* in control and *FaU-box127*-overexpressed fruit. **(C)**, fruit skin color values in control and *FaU-box127*-overexpressed fruit. **(D)**, fruit firmness. **(E)**, the content of pelargonidin 3-glucoside. Data were represented by mean ± standard deviation. The asterisk above the bars indicated the significant difference at *P* ≤ 0.01 level.

To validate the role of FaU-box genes in strawberry fruit ripening, one FaU-box gene (FaU-box127) included in cluster 5 was selected for functional analysis by transiently overexpressing in strawberry fruit. As a result, the control fruit reached the turning stage, while the FaU-box127-overexpressed fruit turned to the full red stage after 5 days infiltration (**Figures 11A, B**). In addition, the a* value was increased, but the L* and b* values were significantly down-regulated in the FaU-box127-overexpressed fruit (**Figure 11C**), indicating the fruit skin was redder and less bright than the control. The fruit firmness was also evidently decreased in the FaU-box127-overexpressed samples (**Figure 11D**). Correspondingly, the content of pelargonidin 3-glucoside (the major anthocyanin in strawberry fruit) was significantly increased in the FaU-box127-overexpressed samples (**Figure 11E**). All these results revealed that the FaU-box127 promoted the ripening of strawberry fruit.

**Figure 12:**
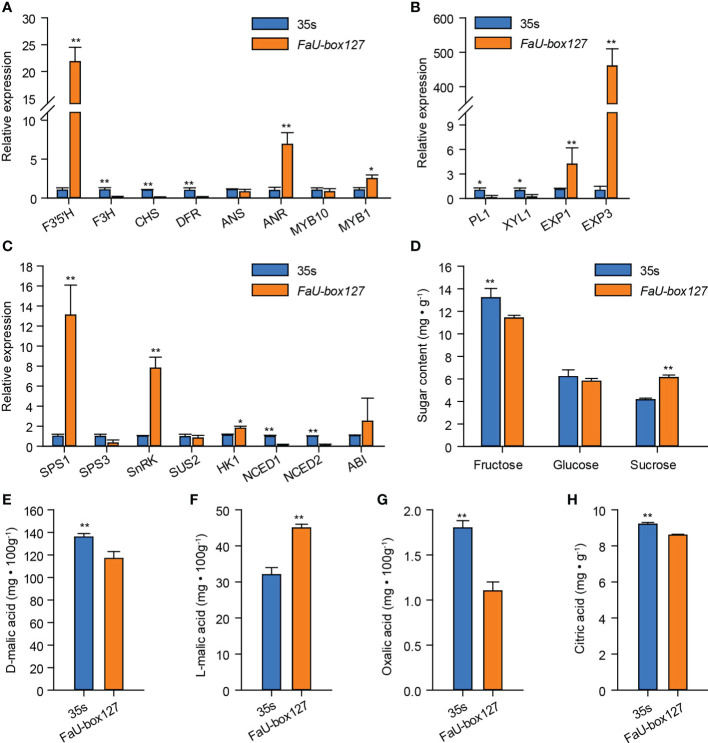
The expression of ripening-related genes and changes of organic acids and sugars content in *FaU-box127*-overexpressed fruit. **(A)**, relative expression of anthocyanins-related genes; **(B)**, relative expression of key genes related to fruit firmness; **(C)**, relative expression of key genes involved in sucrose and ABA metabolism; **(D)**, the changes of soluble sugars; **(E-H)**, the content of D-malic acid, L-malic acid, oxalic acid, and citric acid. Data were represented by mean ± standard deviation. The asterisk above the bars indicated the significant difference at *P* ≤ 0.01 level (**) or *P* ≤ 0.05 level (*).

The expression levels of various ripening-related genes were estimated. As the results showed, among the anthocyanins-related genes, overexpression of *FaU-box127* largely increased the expression of *F3’5’H*, ANR, and MYB1, while decreasing the F3H, CHS, and DFR expression (**Figure 12A**). No significant change in ANS and MYB10 expression was observed in *FaU-box127*-overexpressed fruit compared to the control. Furthermore, two key genes (EXP1 and EXP3) related to fruit firmness were sharply increased by *FaU-box127* overexpressing, especially the EXP3, whose expression level was increased to over 300 times higher than that in the control fruit (**Figure 12B**). In addition, three key genes involved in sucrose metabolism namely SPS1, SnRK, and HK1 were up-regulated in expression (**Figure 12C**). There was no evident change in SPS3, SUS2, and ABI expression. However, the expression levels of NCED1 and NCED2, key genes involved in ABA biosynthesis, were lower in *FaU-box127*-overexpressed fruit than that in the control fruit (**Figure 12C**). These results suggested that the *FaU-box127* overexpression could alter the expression of ripening-related genes.

In general, fruit ripening occurs accompanied by a decrease in organic acids while an increase in sugars. According to our results, the organic acids including D-malic acid, oxalic acid, and citric acid contents were significantly decreased in the *FaU-box127*-overexpressed fruit, (**Figures 12E, G, H**). On the contrary, the content of L-malic acid was higher in the *FaU-box127*-overexpressed fruit than that in the control (**Figure 12F**). In addition, the sucrose content was significantly increased by *FaU-box127* overexpression, while the fructose content showed the opposite trend (**Figure 12D**). The content of glucose exhibited no obvious change in FaU-box127-overexpressed and control fruit.

## 4 Discussion

*U-box* genes belonging to the E3 ubiquitin ligases family are widely distributed in plants. Their characteristics and functions have been comprehensively studied in several plant species, such as *Arabidopsis (* Wiborg et al., 2008), tomato (Sharma and Taganna, 2020), and pear (Wang et al., 2021). However, the knowledge and analysis related to *U-box* genes were poor in cultivated strawberries, which are one of the most important and popular economic fruit trees all over the world. In the present study, we have identified a total of 155 *U-box* genes in the cultivated strawberry genome. This number is much greater than that of *Arabidopsis thaliana* (63), tomato (62), banana (91) (Hu et al., 2018), and pear (62) (Wang et al., 2021), indicating an expansion of this family in the strawberry genome. Our subsequent duplication modes analysis suggested that WGD/segmental duplication was the main driving force for *U-box* gene family expansion in strawberries rather than tandem, singleton, proximal, or dispersed (**Supplementary Table S1**). This duplication apparently derived from the recent whole-genome duplication events in the strawberry genome (Edger et al., 2019), which is similar to that in tomato (Sharma and Taganna, 2020), revealing a comparative common non-random conserved duplication modes in different species (Wang et al., 2011). Furthermore, the Ka/Ks ratios were estimated and the results showed that most of the paralogous pairs among strawberry/*Arabidopsis* and strawberry/woodland strawberry comprised a Ka/Ks ratio of less than one (**Supplementary Table S4**), suggesting they were retained by negative selection (Hurst, 2002). On the contrary, three pairs experienced positive selection with a ratio of Ka/Ks greater than one (**Supplementary Table S4**).

According to the phylogenetic tree analysis, it was evident that 155 *FaU-box* genes were clarified into 4 groups (**Figure 3**), which is similar to other species (Hajibarat et al., 2022). All four groups showed good similarity among themselves due to the presence of the U-box domain, which was a protein-protein interaction domain in E3 ubiquitin ligases in general (Patterson, 2002). In addition to the U-box domain, the FaU-box proteins were found to contain many other domains including ARM repeats, PK_Tyr_Ser-Thr, PKinase, KAP, and WD40 domains (**Figure 3**). It has been suggested that the ARM repeats primarily mediated the interaction of U-box proteins and their substrates, making the substrates available for ubiquitination (Wang et al., 2020). What’s more, the great majority of *U-box* genes that have been demonstrated for biological functions were largely derived from these U-box proteins possessing ARM repeats (Shu and Yang, 2017). In our results (**Figure 3**), 70 FaU-box proteins only possessed the U-box domain, and 61 members contained both U-box and ARM domains. These differentially distributed domains in the FaU-box family indicated their probable divergent biological functions.

Studies have shown that *U-box* genes play important roles in regulating plant growth and development. For example, *AtPUB43* and *PUB44* participate in seed germination and early seedling growth (Salt et al., 2011), while *AtPUB25* and *PUB26* are suggested as important regulators of organ growth (Li et al., 2021). Based on analysis of the *cis*-acting elements presented in the promoters, our results suggested that multiple *FaU-box* genes contained the AACA-motif involved in endosperm-specific, motif I associated with root-specific, and RY-element involved in seed-specific regulation in their promoters (**Supplementary Table S5**, **Figure S4**). These results revealed the putative involvement of *FaU-box* genes in strawberry growth and development. Recently, it has been demonstrated that *U-box* genes may function in the regulation of ripening in peaches (Tan et al., 2019) and grapes (Yu et al., 2020). Here, the potential roles of *U-box* genes in strawberry fruit were estimated by analyzing the expression patterns during fruit development and ripening and under the treatment of ABA and sucrose, which were previously proven to coordinately promote strawberry fruit ripening (Luo et al., 2020). The results (**Figure 6**) showed that most of the *FaU-box* genes were highly expressed in the early developmental stage (LG) while lowly expressed in the late ripening stage (FR). This is consistent with that in grapes (Yu et al., 2020) and bananas (Hu et al., 2018), indicating the similar function of these genes. Moreover, there were 23 *FaU-box* genes increased with fruit ripening in expression (**Figure 6**), which revealed the potential positive regulatory roles in strawberry fruit ripening. Furthermore, since their expression gradually decreased during strawberry fruit ripening (**Figure 7**), *FaU-box9*, *FaU-box52*, and *FaU-box93* were suggested negatively related to strawberry ripening. Combined with the results that no significant difference for the expression of *FaU-box9* and *FaU-box93*, but significant repression of *FaU-box52* expression under the treatment of a mixture of ABA and sucrose, *FaU-box52* was identified as a candidate negative regulator FaU-box member of strawberry fruit ripening. On the contrary, the expression of *FaU-box98* and *FaU-box136* increased with fruit ripen as showed by both RNA-seq and RT-qPCR based expression analysis (**Figures 6**, **7**), and their expression levels were significantly up-regulated by ABA and sucrose treatment. Therefore, it is reasonable to speculate that *FaU-box98* and *FaU-box136* were activators involved in strawberry fruit ripening. Eventually, based on the RT-qPCR results, we found that the expression of *FaU-box142* was higher in the late developmental stage than early stages (**Figure 7A**), and also largely up-regulated by ABA and sucrose treatment (**Figure 7B**), indicating *FaU-box142* was also positively associated with strawberry fruit ripening. However, this result was opposite to the RNAseq result (**Figure 6**), which needs further research. In addition, some reports have shown that U-box genes that are decreasingly expressed during the fruit development stage are also associated with ripening. For example, grapevine U-box E3 ubiquitin ligase VlPUB38 negatively regulates fruit ripening through the ABA pathway (Yu et al., 2020). We randomly selected one *FaU-box* (*FaU-box127*) among the identified *FaU-box* genes for functional analysis. Transient overexpression of *FaU-box127* in strawberry fruits resulted in increased anthocyanins, sucrose, and L-malic acid, but decreased brightness, fruit firmness, D-malic acid, oxalic acid, citric acid and fructose (**Figures 11**, **12**). Meanwhile, the expression levels of some ripening-related genes were significantly increased or decreased. Based on previous reports (Yu et al., 2020; Li et al., 2022), we speculated that FaU-box127 may regulate fruit ripening by affecting the ABA or sucrose pathways, but the mechanism is still unclear and it needs further study.

Additionally, previous reports have illustrated that *U-box* genes can respond to ABA signals. However, the changes in *U-box* gene expression differed with species in response to ABA treatment. In tomatoes, no major expression difference was observed among the *U-box E3* genes under ABA treatment (Sharma and Taganna, 2020). Whereas, significant induction of *PalPUB79* expression by ABA signaling was found in Populus (Tong et al., 2021). In this study, a lot of ABA-responsive elements were presented in the putative promoter regions of various *FaU-box* genes (**Supplementary Table S5**, **Figure S4**), and the ABA-insensitive protein ABI1 was involved in the interaction of FaU-box genes (**Figure 5**). In support of this, the subsequent expression analysis showed several *FaU-box* genes were significantly induced or repressed by exogenous ABA treatment (**Figure 8**). Of these, *FaU-box3*, *FaU-box65*, and *FaU-box142* were suggested to be negative while *FaU-box18*, *FaU-box52*, and *FaU-box98* were positive responses to ABA. All these results implied the potential participation of *FaU-box* genes in the ABA signaling pathway. ABA has been commonly regarded as the key regulator of strawberry fruit ripening (Jia et al., 2011; Jia et al., 2013), and also a stress hormone participating in extensive stress resistance (Sah et al., 2016). Therefore, it is reasonable to speculate that, the potential involvement of *FaU-box* genes in strawberry fruit development and ripening and stress resistance as suggested in this study, might be associated with the participation of *FaU-box* genes in ABA signaling.

Abiotic stresses including cold, drought, and salinity negatively affect the growth and general development of a plant, leading to a huge crop yield penalty worldwide (He et al., 2018). To survive and sustain their growth, plants have therefore developed sophisticated strategies to rapidly respond to changes in their environment. The ubiquitin-proteasome pathway has been suggested as an important way, and a large number of E3 ubiquitin ligases have been reported to function in response to abiotic stresses (Yee and Goring, 2009; Al-Saharin et al., 2022). *AtPUB22/AtPUB23* are negative regulators mediating drought responses (Seo et al., 2012), while *AtPUB25* and *AtPUB26* participated in plant response to low-temperature (Wang et al., 2019). In this study, the differential expression levels of 10 *FaU-box* genes under various abiotic stresses were investigated. According to the result (**Figure 10**), all the detected *FaU-box* genes were significantly up-regulated expressed under drought and salt treatment at 3 and 6 h separately, suggesting the *FaU-box* genes might positively regulate the response process of dehydration and salinity. However, some *FaU-box* genes expression continuously increased until 24 h after treatment, such as *FaU-box83* and *FaU-box136* under drought stress and *FaU-box83*, *FaU-box3, FaU-box98, FaU-box136*, and *FaU-box136* under salt stress. This result suggested that these *FaU-box* members may play major roles in regulating drought and salt stress of strawberry plant. In addition, various *FaU-box* genes were rapidly and largely up-regulated expressed under cold stress, *FaU-box98* was the largest increased one (**Figure 9**). All these results suggested the potential involvement of *FaU-box* genes in response to different abiotic stresses.

## Data availability statement

The original contributions presented in the study are included in the article/**Supplementary Material**. Further inquiries can be directed to the corresponding authors.

## Author contributions

Conceptualization: LJ, YXL, HT, and YL. Methodology: LW and YP. Software, QC, ML, MY, and GH. Validation: XL and YJ. Formal analysis: YXL. Resources: HT and YL. Writing—original draft preparation: LJ. Writing—review and editing: YXL, YTZ, YW, WH, YZ, and XW. Supervision: HT and YL. Funding acquisition: YL. All authors contributed to the article and approved the submitted version.
