# Supplementary figures and images for: Genome-wide identification and expression profiling reveal the regulatory role of U-box E3 ubiquitin ligase genes in strawberry fruit ripening and abiotic stresses resistance
Source: Front Plant Sci. 2023 Mar 23;14:1171056. doi: 10.3389/fpls.2023.1171056 (PMC10078948; doi:10.3389/fpls.2023.1171056)

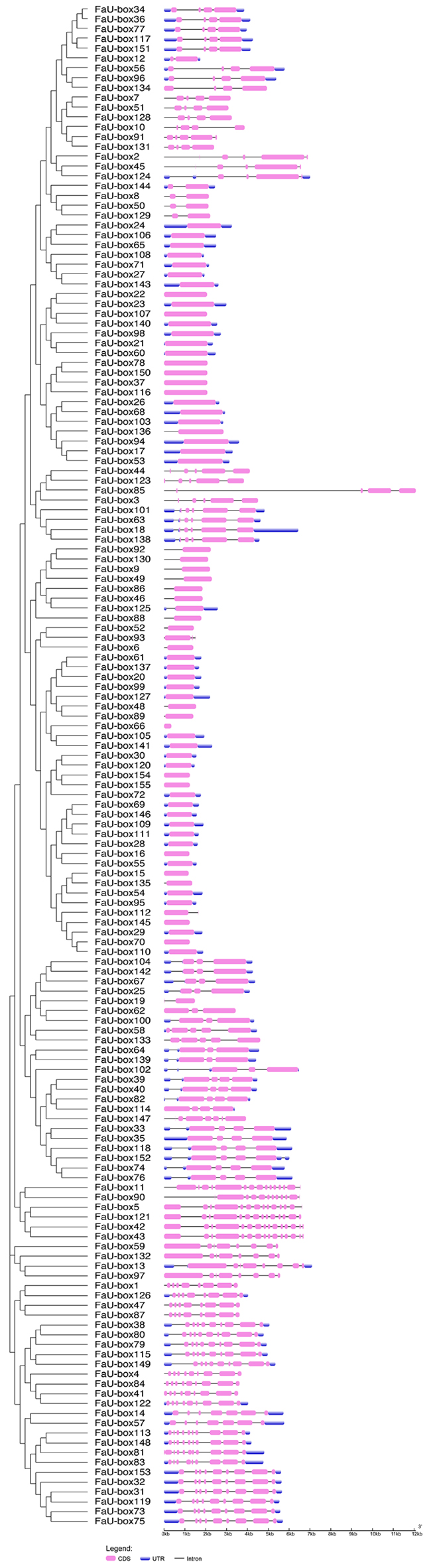

Supplement: Supplementary file 1 [file Image_1.jpeg]

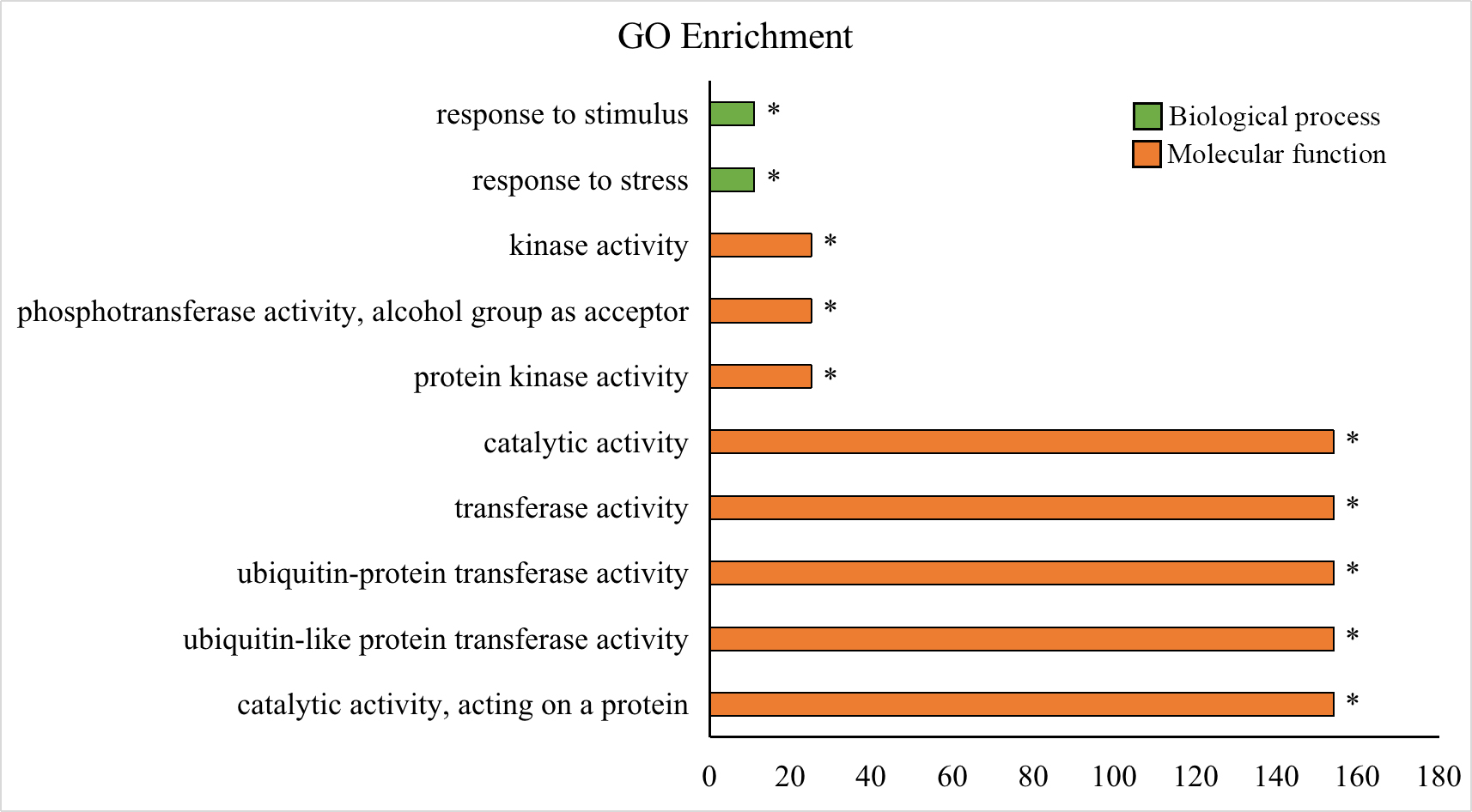

Supplement: Supplementary file 2 [file Image_2.jpeg]

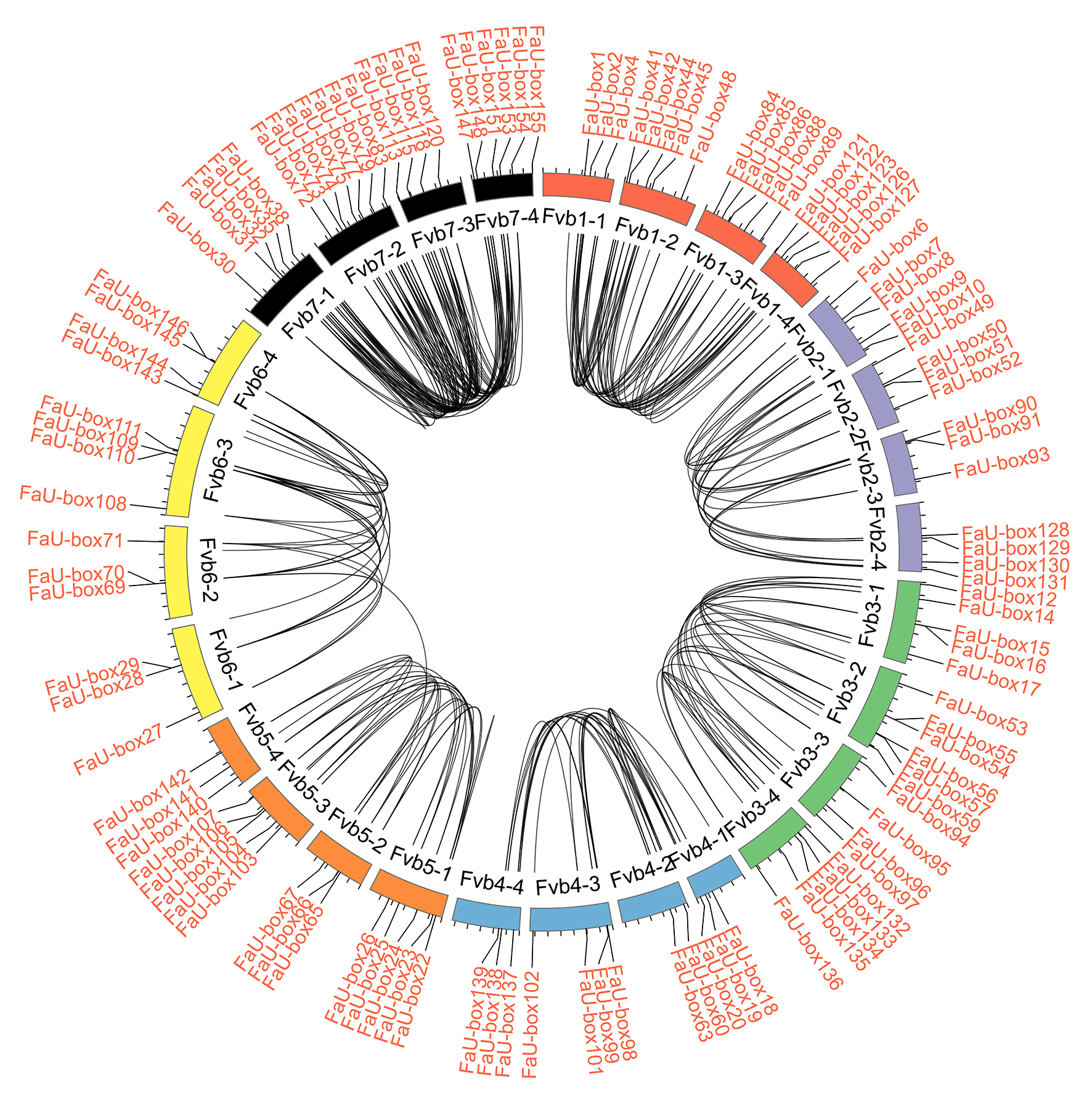

Supplement: Supplementary file 3 [file Image_3.tif]

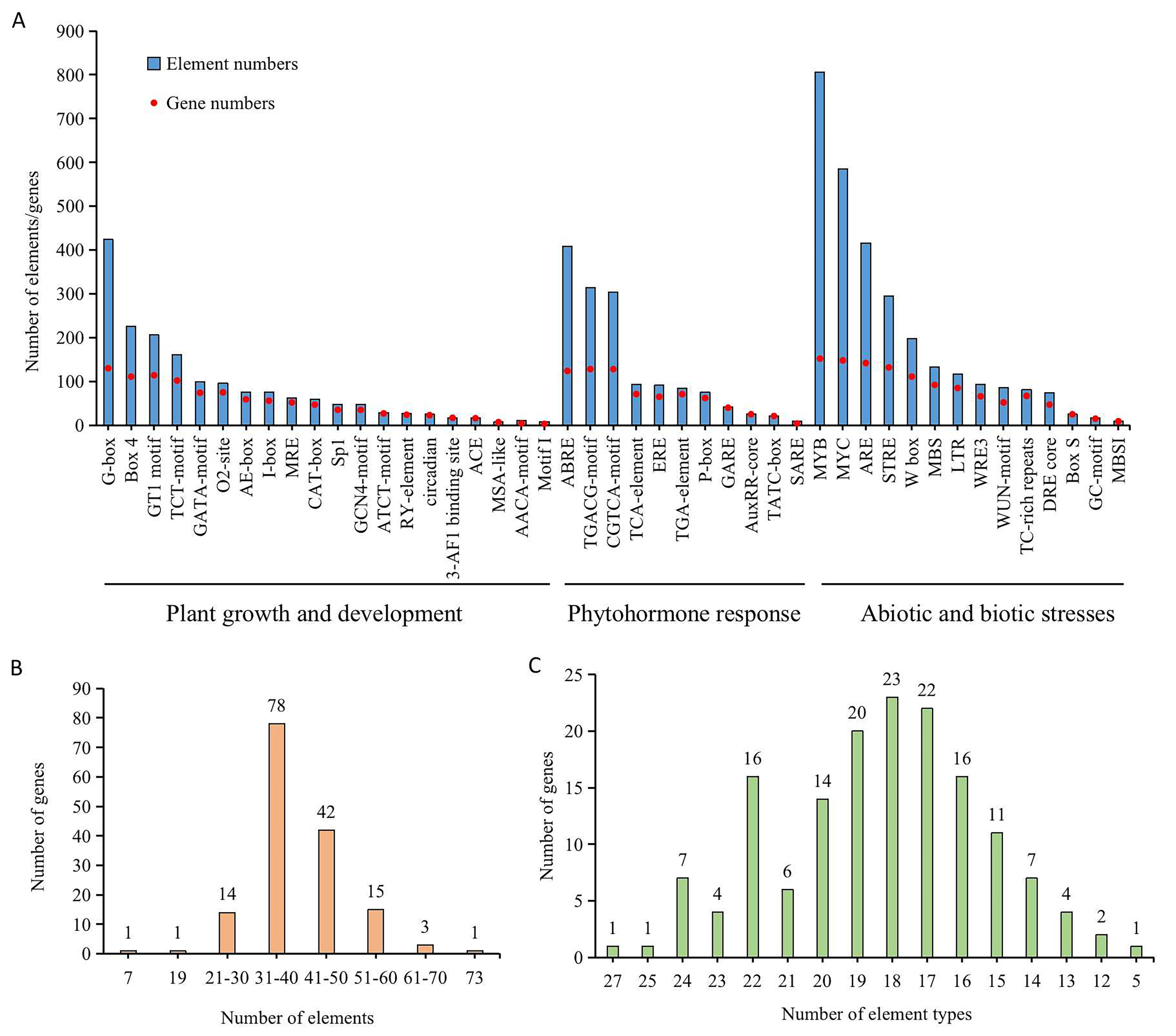

Supplement: Supplementary file 4 [file Image_4.jpeg]

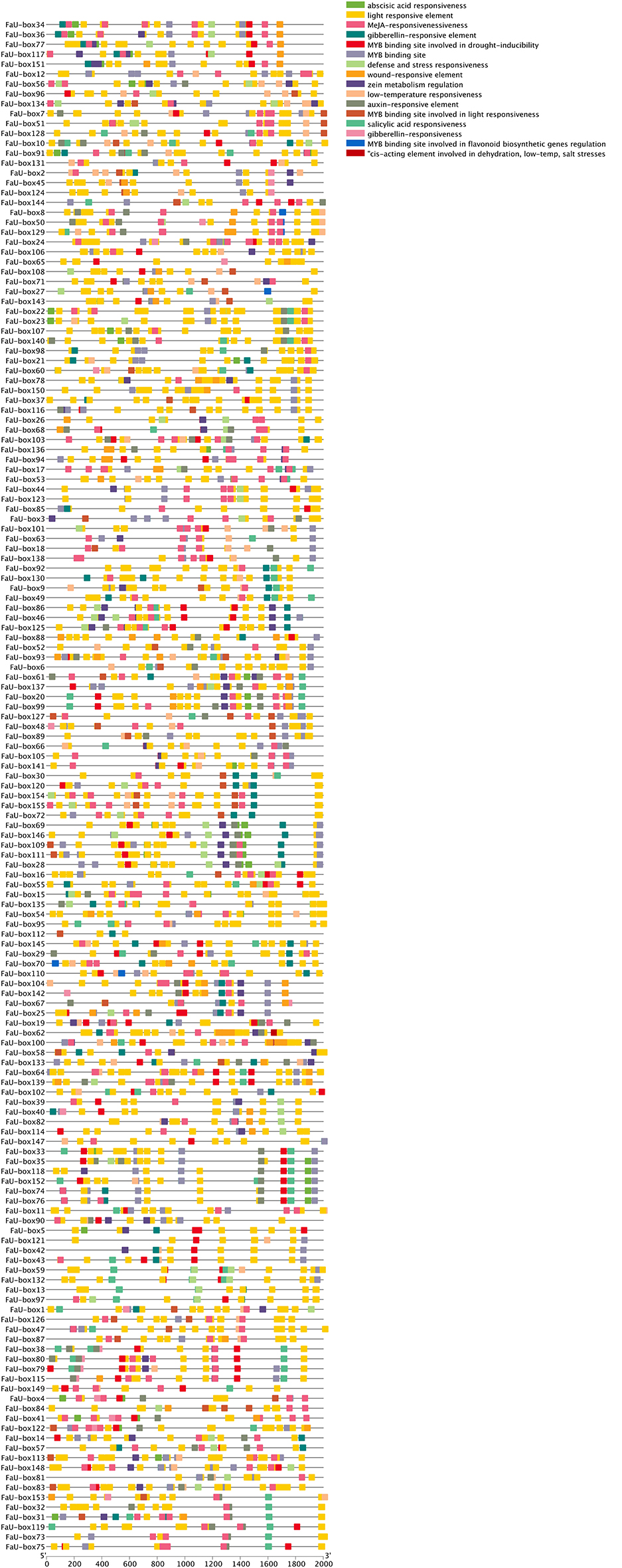

Supplement: Supplementary file 5 [file Image_5.jpeg]
